# Metabolic Response of the *Lycium barbarum* Variety ‘Ningqi No. 7′ to Drought Stress

**DOI:** 10.3390/plants13141935

**Published:** 2024-07-14

**Authors:** Xiao Liu, Chuanzhe Wang, Qiao Xu, Dan Zhao, Fei Liu, Beibei Han

**Affiliations:** 1 College of Resources and Environment, Xinjiang Agricultural University, Urumqi 830052, China; 320223543@xjau.edu.cn (X.L.); 320223547@xjau.edu.cn (C.W.); 254782534@xjau.edu.cn (Q.X.); zd-zhxy@xjau.edu.cn (D.Z.); 320223576@xjau.edu.cn (F.L.); 2 College of First-Year Students, Xinjiang Agricultural University, Urumqi 830052, China

**Keywords:** *Lycium barbarum*, UPLC-MS, differential metabolite, drought resistance mechanism

## Abstract

*Lycium barbarum* has been widely planted in arid and semi-arid areas due to its drought-resistant ability, which is of great economic value as a medicinal and edible homology plant. In this study, the metabolome of the *L. barbarum* variety “Ningqi 7” under different drought stress conditions was compared and analyzed by the non-targeted UPLC-MS (ultra-high performance liquid chromatography with mass spectrometry) technique. The results showed that drought stress significantly decreased the water content of leaves, increased the activity of antioxidant enzymes in plants, and up-regulated the metabolites and pathways involved in osmoregulation, antioxidant stress, energy metabolism, and signal transduction. Under moderate drought (40–45% FC), *L. barbarum* accumulated osmoregulatory substances mainly through the up-regulation of the arginine metabolism pathway. At the same time, phenylalanine metabolism and cutin, suberine, and wax biosynthesis were enhanced to improve the antioxidant capacity and reduce water loss. However, in severe drought (10–15% FC), *L. barbarum* shifted to up-regulate purine metabolism and lysine degradation and redistributed energy and nitrogen resources. In addition, vitamin B6 metabolism was significantly upregulated in both groups of stress levels, playing a key role in antioxidant and growth regulation. These observations delineate the metabolic adaptations of *L. barbarum* “Ningqi 7” in response to drought stress.

## 1. Introduction

*Lycium barbarum* is a perennial deciduous shrub, a medicinal and food homology plant, which has been recorded in traditional Chinese medicine books throughout the ages and is naturally distributed in western China [1]. At present, *L. barbarum* is an important cash crop planted in arid and semi-arid areas of northwest China [2]; water is the main factor restricting plant productivity in these areas [3]. *L. barbarum*, while not a quintessential desert flora, exhibits a notable capacity for drought endurance [4]. This resilience is primarily attributed to its robust root system, capable of extracting moisture from deeper soil strata during arid periods [5]. Additionally, its small leaf surface area minimizes water transpiration, thereby conserving hydration. The plant’s adaptability to diverse soil conditions, including sandy terrains, furthers its potential for cultivation in semi-arid regions [6]. However, under harsh environmental stress, this resistance is very limited. Therefore, more in-depth understanding of *L. barbarum’s* response to water deficit and elucidating physiological countermeasures to adapt to stress is particularly important for screening drought-tolerant varieties or genotypes to combat such constraints.

In recent decades, global climate change has led to an increasing incidence and severity of drought stress in many regions, especially in arid and semi-arid regions [7]. Drought stress leads to insufficient soil moisture in agriculture and forestry production, which seriously reduces the yield and quality of agriculture and forestry products and then affects the regional economy [8]. For *Lycium* plants, studies have shown that drought stress can reduce their ground diameter, seedling height, shoot length, shoot thickness, leaf fresh weight, leaf dry weight, and leaf area [9,10]. With the increase in water deficit, the branch growth rate is slow, chlorophyll content is reduced, and the dry matter allocation rate in stems is gradually increased, while the dry matter allocation rate in branches, leaves, and fruits is significantly reduced [11,12]. Persistent drought also inhibits the accumulation of fructose and sucrose in ripening fruits [13]. Studies on *Lycium* under drought conditions focused on the physiological indexes of plant growth and the accumulation of effective components in fruits [14,15]. However, the mechanism of *Lycium*’s response to drought stress, especially its adaptability at the molecular level, was very limited.

Therefore, we selected Ningqi No. 7, a new *L. barbarum* variety developed in recent years, which is widely cultivated in Jinghe County, Bortala Mongolian Autonomous Prefecture, Xinjiang, China. It is a geographical indication of agricultural products and a Sino-European mutual recognition product [16]. The production and cultivation of *L. barbarum* is the leading industry with the most local characteristics and brings great economic benefits to the region. In this study, the metabolome of “Ningqi No. 7” under normal water supply and drought stress was compared and analyzed by means of non-targeted UPLC-MS. The aim was to explore the metabolic changes in *L. barbarum* and other crops under drought stress, identify the key metabolites and pathways related to drought tolerance, and reveal the metabolic mechanism of *L. barbarum* and other crops under drought stress, so as to provide a theoretical basis for drought-resistant breeding.

## 2. Results

### 2.1. Changes in the Physiological Indices of L. barbarum under Drought Stress

**Figure 1:**
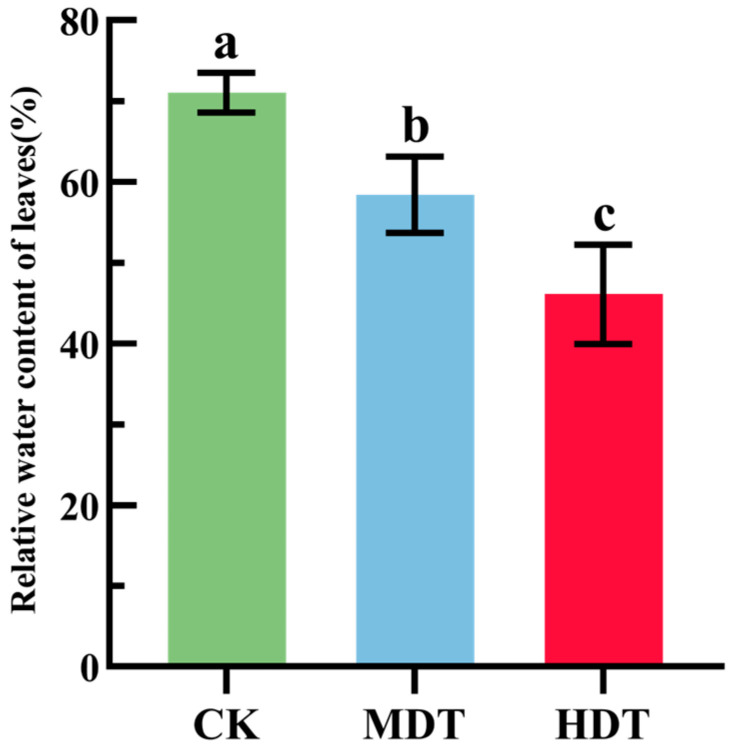
Changes in the water content of *L. barbarum* leaves under drought stress; different lowercase letters indicate significant differences *p* < 0.05.

Leaf relative water content (RWC) is the most direct index to measure the water status of plants. As shown in Figure 1, there were significant differences in RWC among the control group (CK), moderate drought treatment (MDT), and severe drought treatment (HDT) treatments (*p* < 0.05). RWC in the MDT and HDT groups decreased by 12.61% and 26.93% compared with the CK group, respectively. The results showed that the plants were subjected to drought stress in different degrees by water control.

**Figure 2:**
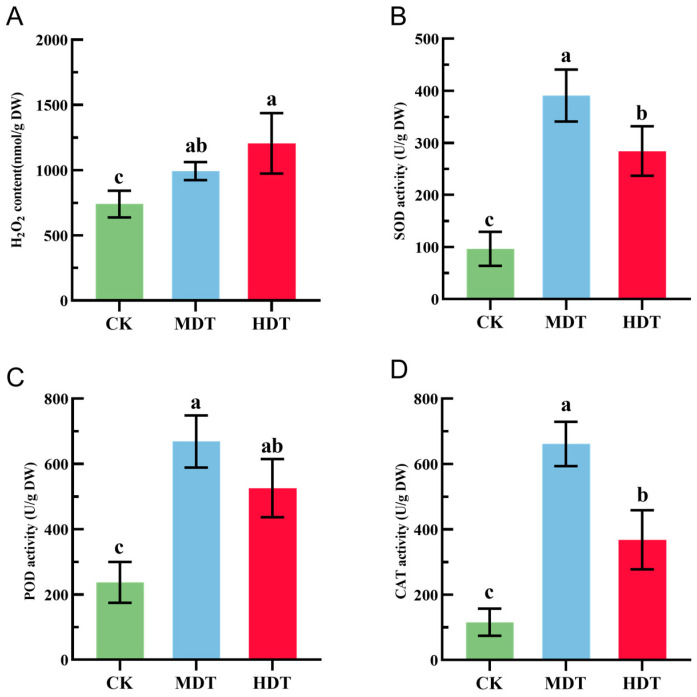
H_2_O_2_ content (**A**), SOD activity (**B**), POD activity (**C**), and CAT activity (**D**) of *L. barbarum* leaves under drought stress; different lowercase letters indicate significant differences *p* < 0.05.

When plants are stressed, it causes the outbreak of Reactive Oxygen Species (ROS) in plants; excessive ROS will cause damage to cell structure and function. Plants reduce the damage to cells by increasing the activity of antioxidant enzymes. As shown in Figure 2, the H_2_O_2_ content of *L. barbarum* plants under MDT and HDT drought stress treatments was significantly higher than that of the control group (*p* < 0.05), most notably it was the highest in the HDT group, in which it was 62.81% higher than that in CK. With the intensification of stress, the content of H_2_O_2_ in plants showed an increasing trend. The activities of SOD, POD, and CAT in the two groups under drought stress were significantly higher than those in CK (*p* < 0.05). However, the activities of the three enzymes in the HDT group were decreased compared with those in the MDT group and the activities of SOD and CAT were significantly lower than those in the MDT group (*p* < 0.05). These results indicate that *L. barbarum* plants have a limited ability to clear excess ROS by increasing antioxidant enzyme activity when stress intensification occurs. It may be due to the severe stress caused by serious damage to the plant itself, which reduces the ability to regulate the activity of antioxidant enzymes. Since there are complex metabolic processes in plants, the physiological processes surrounding ROS are only part of it. It is also possible that under severe stress, plants activate more complex metabolic activities to resist stress.

### 2.2. Metabolite Statistics and Quality Control

Because the chromatographic system and mass spectrometry are in direct contact with the sample, with the increase in the analyzed sample, the chromatographic column and mass spectrometry will gradually pollute, resulting in signal drift and systematic error of measurement. The repeatability and accuracy of the measured data are corrected by repeatedly using the same Quality Control (QC) samples to track the entire data collection process [17]. QC is the sample after an equal amount of all samples are mixed. During the collection process, QC is inserted after every three test samples to record the signal drift. After correcting signal drift, QC sample points were gathered together (Figure S1), which proved that the correction effect was good and the data were available.

A total of 12,212 peaks and 1357 metabolites were detected from the positive ion mode in the control group and the two drought stress treatment groups; 11,688 peaks and 605 metabolites were detected in the negative ion mode.

### 2.3. Principal Component Analysis

**Figure 3:**
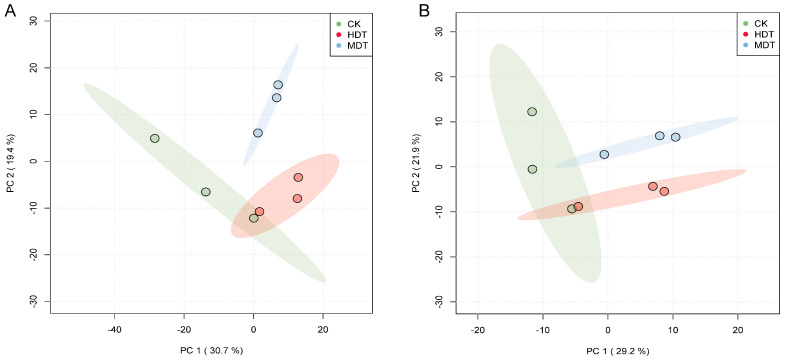
PCA results of metabolites in *L. barbarum* leaves. Positive ion mode (**A**); Negative ion mode (**B**).

Principal component analysis (PCA) was used to compare intergroup metabolic differences in MDT, HDT, and CK to determine the overall metabolomic changes in *L. barbarum*; the reproducibility and stability of the metabolites were evaluated. Figure 3A and Figure 3B, respectively, show the PCA results of the three groups of samples in positive and negative ion modes. The total interpretability shown in the two figures is 50.1% and 51.1%, respectively, which can better show the clustering of samples. Although the three groups have some overlap, they still show obvious differences, indicating that drought stress has significant effects on *L. barbarum* plant metabolism and that the effects on plant metabolism are different from the aggravation of drought degree.

### 2.4. Orthogonal Partial Least Squares Discriminant Analysis

**Figure 4:**
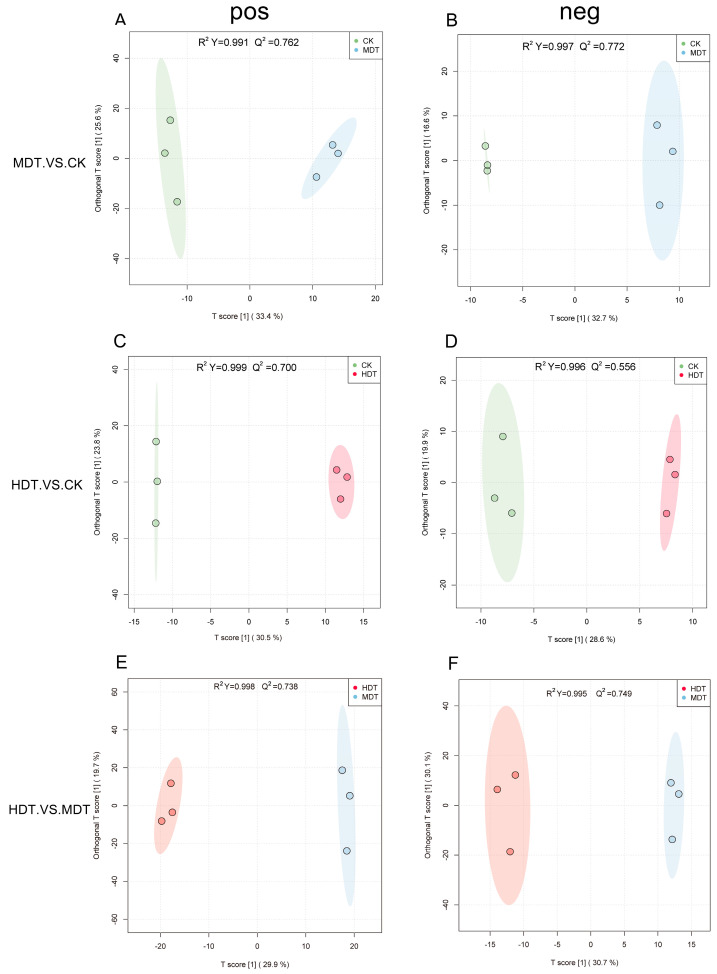
OPLS-DA results of *L. barbarum* leaf metabolites. Positive ion mode (**A**,**C**,**E**); Negative ion mode (**B**,**D**,**F**).

Groups HDT, MDT, and CK were pairwise for OPLS-DA analysis (Figure 4). The results show that under the positive and negative ion modes, there is a very obvious difference between HDT and MDT and between the samples of the two groups of drought treatment and the samples of the CK group, respectively. The R^2^Y and Q^2^ of each comparison group were all greater than 0.99 and 0.5, respectively, indicating that the model had a good distinction effect [18]. The differential metabolites can be screened according to VIP value analysis.

### 2.5. Screening of Differential Metabolites

**Figure 5:**
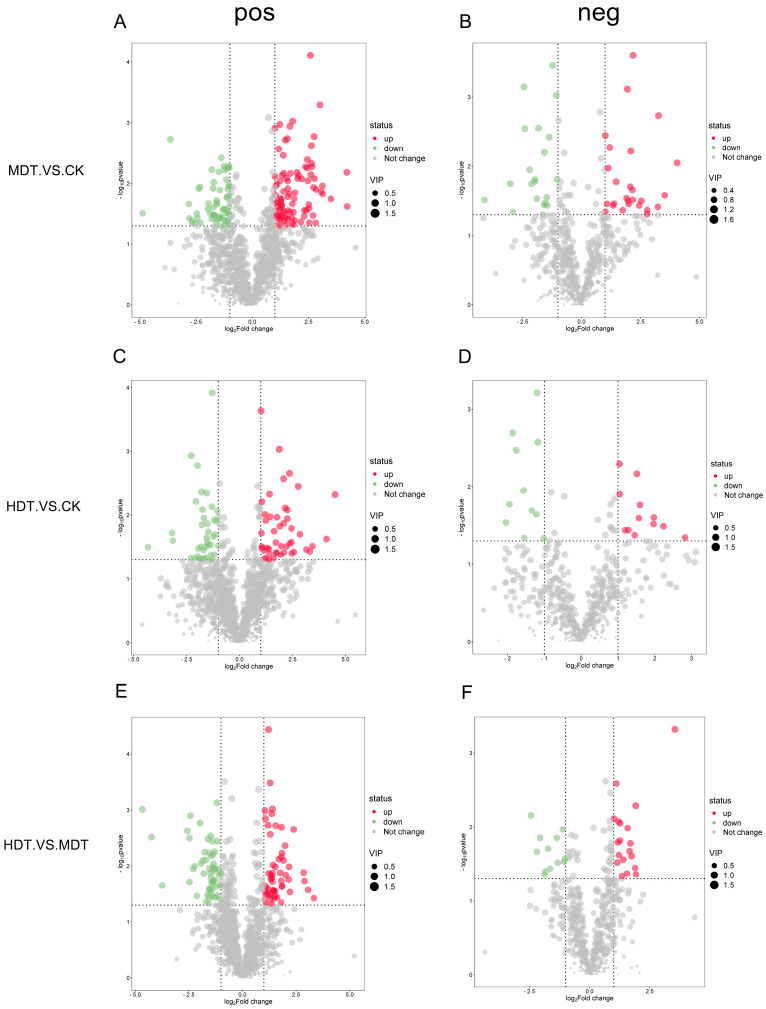
Volcano map of differential metabolites combined with VIP values. Positive ion mode (**A**,**C**,**E**); Negative ion mode (**B**,**D**,**F**).

**Figure 6:**
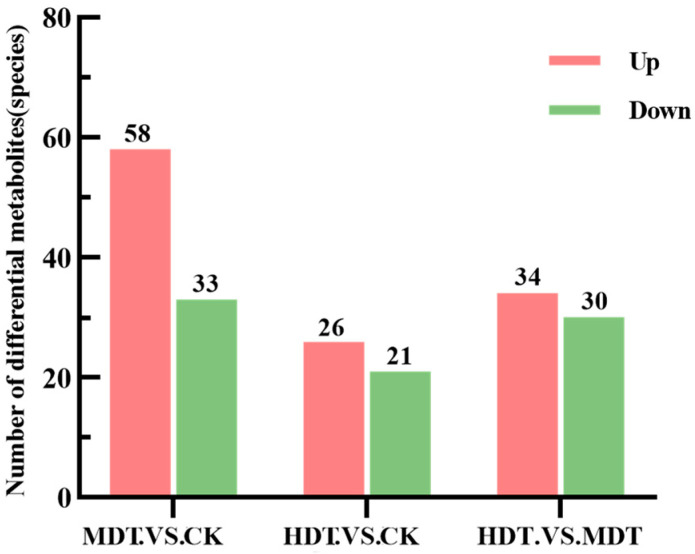
The number of differentiated metabolites between groups.

**Figure 7:**
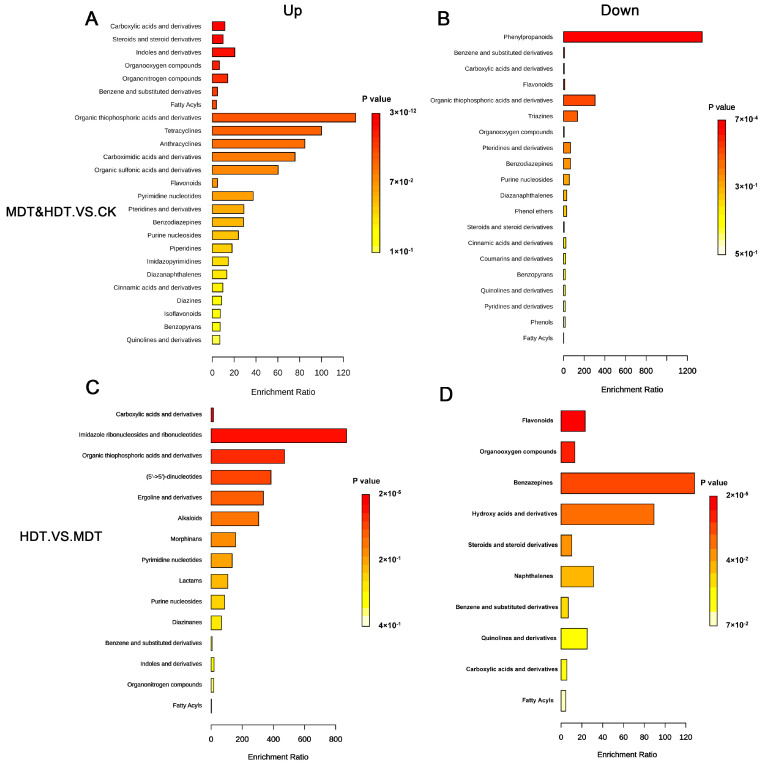
Enrichment analysis of differential metabolites according to chemical structures. Up-regulated group(**A**,**C**); Down-regulated group (**B**,**D**).

We performed a differential analysis of metabolites. As shown in Figure 5, the screening criteria were log2(FoldChange) ≥ 1 and *p*-value < 0.05. Combined with the screening results of VIP (Variable Importance in Projection) > 1 in the OPLS-DA model, the final differential metabolites were obtained. To make the statistics more biologically significant, we used differential metabolites with KEGG (Kyoto Encyclopedia of Genes and Genomes) database ids for analysis. As shown in Figure 6, after combining the compounds in the positive and negative ion mode, 58 metabolites in the MDT were up-regulated and 33 were down-regulated compared with CK; 26 up-regulated and 21 down-regulated compared with CK in the HDT. The differential metabolites were enriched according to the compound structure. Compared with the control, carboxylic acids and derivatives, steroids and steroid derivatives, and Indoles and derivatives were upregulated in the two stress treatments (Figure 7A). Down-regulated compounds were phenylpropanoids, flavonoids, etc. (Figure 7B). When the HDT and the MDT were compared separately, 34 kinds of metabolites in the HDT were up-regulated (Figure 7C). They are mainly carboxylic acids and derivatives, imidazole ribonucleosides and ribonucleotides, and alkaloids. There are 30 compounds in MDT (Figure 7D), which are mainly flavonoids and organooxygen compounds. The results showed that drought stress caused great changes in leaf metabolism of *L. barbarum* plants compared with control plants and, with the aggravation of stress, *L. barbarum* constantly adjusted metabolic strategies to cope with stress.

### 2.6. KEGG Pathway Enrichment Analysis of Differential Metabolites

**Figure 8:**
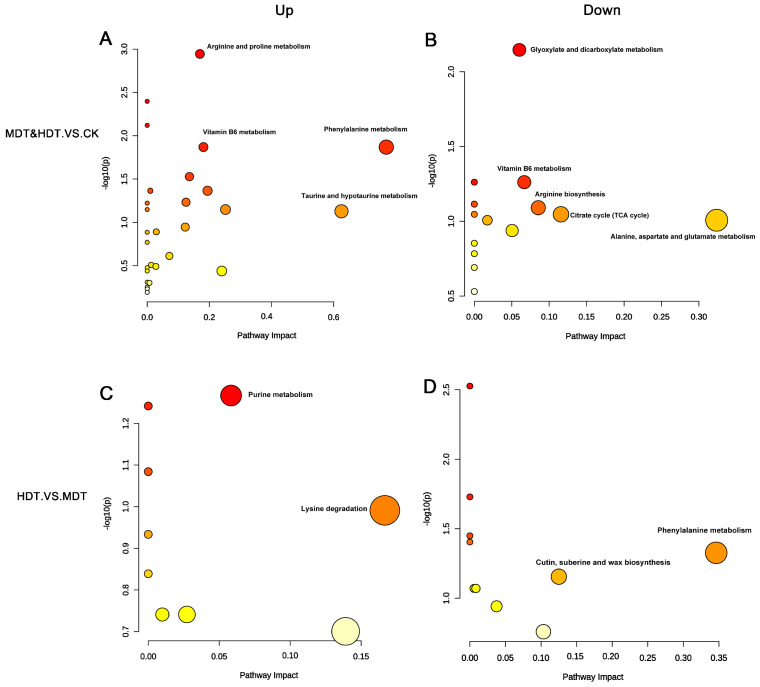
Metabolic pathway analysis of differential metabolites based on the KEGG database. Up-regulated group(**A**,**C**); Down-regulated group (**B**,**D**).

We performed an enrichment analysis of metabolic pathways for differential metabolites with ID through the KEGG database (Table S1). We found that compounds upregulated in the two stress treatment groups compared with the control group were mainly enriched in the following pathways (Figure 8A): arginine and proline metabolism, vitamin B6 metabolism, phenylalanine metabolism, and taurine and hypotaurine metabolism. The main role of these pathways may be to participate in osmoregulation to maintain intracellular water balance and enhance the protective mechanism against oxidative stress [19]. The primary pathways exhibiting downregulation in *L. barbarum* under drought stress encompass glyoxylate and dicarboxylate metabolism, arginine biosynthesis, alanine metabolism, aspartate and glutamate metabolism, and the citrate cycle (Figure 8B). The attenuation of these metabolic processes implies a concerted effort by *L. barbarum* to curtail energy expenditure to the greatest extent feasible during periods of water deficit [20]. At the same time, we noticed that vitamin B6 metabolism and arginine biosynthesis appeared in both the up-regulated and down-regulated pathways, indicating that these two pathways may intersect with other metabolic pathways and can be used as key pathways for further analysis. When the HDT and the MDT were compared separately, the up-regulated pathways in the MDT were mainly as follows: phenylalanine metabolism and cutin, suberine, and wax biosynthesis (Figure 8D) showed that *L. barbarum* plants could actively respond to moderate stress to enhance their protective mechanisms [21]. The main up-regulated pathways in the HDT are purine metabolism and lysine degradation (Figure 8C), indicating that *L. barbarum* plants may adjust energy metabolism under severe stress and degrade lysine for nitrogen recovery to resist extreme environments [19,22].

### 2.7. Analysis of Key Metabolic Pathways

**Figure 9:**
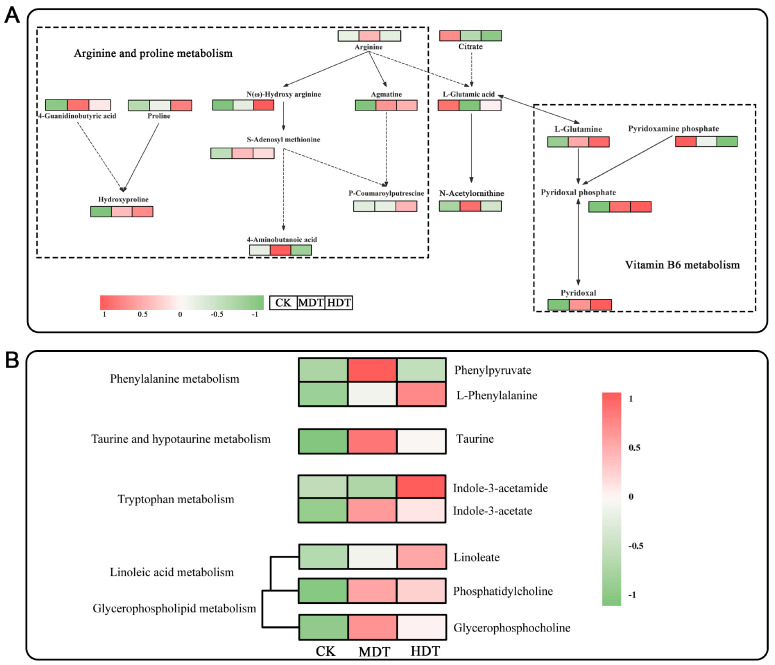
Up-regulated and relatively complete critical pathways in *L. barbarum* leaves under drought stress (**A**). Heat map of the metabolite content of higher importance in the corresponding pathway (**B**). The solid lines represent direct synthesis and the dashed lines represent indirect synthesis or synthetic precursors.

We annotated the content of individual monomer metabolites in the major up-regulated pathways (Figure 9). We noted that L-glutamic acid appears to act as a crossover hub in arginine and proline metabolism and VB6 metabolism (Figure 9A). In addition to affecting the above two pathways, it also involves arginine biosynthesis. Therefore, the downregulation of L-glutamic acid and pyridoxamine phosphate causes VB6 metabolism and arginine biosynthesis to be enriched in both up-regulated and down-regulated pathways. However, the conversion of L-glutamine to pyridoxal phosphate is a major part of the production of the activated form of VB6 [23]. In this stress test, this pathway was upregulated and the reaction was complete as the stress level increased. Therefore, it can be regarded as one of the pathways of *L. barbarum* signature rise under drought stress and its upregulated metabolites can be used as signature metabolites to reflect stress situations. Among the other upregulated primary metabolic pathways, in addition to the conventional plant stress resistance pathways of phenylalanine metabolism and tryptophan metabolism [19], taurine in the taurine and hypotaurine metabolism and linoleate and phosphatidylcholine in the linoleic acid metabolism can also serve as metabolic markers for *L. barbarum* plants to cope with drought stress (Figure 9B).

## 3. Discussion

In previous studies on *Lycium* plants, Zhao et al. [24] found that drought stress affected the chlorophyll content, net photosynthetic rate, and transpiration rate of *Lycium* due to its restriction of water content in plants. Due to the obstruction of photosynthesis, a large amount of ROS is produced in plants, which also increases the degree of lipid membrane peroxidation and activates the activities of four antioxidant enzymes, POD, CAT, SOD, and APX. This is consistent with the results of the activation of the antioxidant enzyme system in *L. barbarum* plants after drought stress in this study. Li et al. [25] found in their study on *L*. *ruthenicum* that moderate salinity (0–100 mM) and low water deficit (75–100% FC) stimulated the photosynthetic rate and stomatal conductivity but high salinity (200–400 mM) and severe water stress (30–60% FC) inhibited the photosynthetic capacity; SOD and POD activities increased significantly with the increase in stress level. This is different from the trend of the enzyme activity in this experiment, which firstly increased and then decreased with the aggravation of stress degree. This may be due to the difference between *Lycium* plants and different species and the difference between stress types and stress levels. In the experiment of *L*. *ruthenicum* seedlings under a single drought stress, Guo [26] found that the activities of antioxidant enzymes SOD, POD, and CAT in leaves first increased and then decreased; the same conclusion was reached under similar stress conditions as in this study.

When plants are subjected to drought stress, the most direct process is that drought leads to stomatal closure, reducing the entry of CO_2_ and reducing the photosynthetic rate [27]. At the same time, the photosynthetic electron transport chain is overexcited due to the unweakened light intensity, resulting in excessive excitation energy. This imbalance will leak electrons to oxygen molecules, forming a superoxide anion radical (O_2_•−) [28]. Therefore, the content of H_2_O_2_ is usually used to reflect the intensity of ROS production [29,30]. In this study, H_2_O_2_ content in *L. barbarum* leaves continued to increase with the intensification of stress (Figure 2A). It shows that free radicals are constantly produced in excess, which leads to lipid membrane peroxidation and osmotic pressure inside and outside the cell [31]. It also puts stress on the antioxidant system, interferes with normal metabolic pathways, and leads to the accumulation of intermediates, which may in turn participate in the production of ROS and ultimately lead to decreased enzyme activity due to cell damage or intracellular resource allocation problems [32]. Therefore, in this study, with the intensification of stress, the activity of antioxidant enzymes showed a trend of first increasing and then decreasing but still maintained a significantly higher activity than CK (Figure 2). The decline in enzyme activity does not mean that plants will immediately die, especially plants with a certain degree of drought tolerance such as *L. barbarum*, which can resist drought stress by synthesizing non-enzymatic antioxidants, accumulating osmoregulatory substances and avoiding growth-regulating metabolic pathways such as overactivation of photosynthesis [33].

In the present study, it was observed that following drought stress, the metabolism of arginine and proline in the leaves of *L. barbarum* was significantly upregulated. Historical research has positioned proline metabolism as a central regulatory hub under stress conditions in plants [34]. Notably, proline, a well-recognized protective compound, is involved in osmotic adjustment within plant cells, influences cell wall formation, and aids in maintaining intracellular osmotic balance during water deficit [35,36]. Arginine, a precursor to polyamines and proline, is metabolized within the plant through arginase (ARGAH), arginine decarboxylase (ADC), and nitric oxide synthase (NOS) [37]. These enzymes also participate in the clearance of ROS and are involved in the synthesis of NO [38], a crucial signaling molecule that modulates the plant’s adaptability to drought stress [39]. Studies have further indicated that arginine metabolism is implicated in the regulation of heat shock proteins (HSPs) and other chaperone genes, which play roles in protecting cells from damage and facilitating the repair of damaged cells [40]. Additionally, it is associated with the synthesis and signaling pathways of plant hormones such as abscisic acid (ABA), a key hormone that regulates the plant’s response to drought stress [41]. In the key pathways discussed in this study, L-glutamic acid in the arginine and proline metabolism appears to act as an intersecting hub, also connecting to VB6 metabolism, arginine biosynthesis, and the citrate cycle. This further confirms that L-glutamic acid is a central molecule in plant amino acid metabolism; yet, due to its versatile roles and diverse functions across various pathways, it is typically not considered a marker metabolite.

However, the conversion of L-glutamic acid to L-glutamine, the synthesis of pyridoxal phosphate (PLP), and the final synthesis of pyridoxal (PL) are a major part of producing the activated form of VB6 [42]. This response was upregulated and intact in *L. barbarum* in this test (Figure 9A). VB6 is a potential singlet oxygen quenching agent; its quenching ability is comparable to vitamin C and vitamin E and, even higher than the latter two, the product of its metabolic process can also be used as a growth regulator to stimulate the growth and development of plants [43]. Lu et al. [44] found in their study on the regulation of salt tolerance of maize roots by VB6 that PLP is an antioxidant and coenzyme produced in VB6 metabolism and can reduce excess ROS and regulate ABA biosynthesis, helping maize roots better adapt to salt stress. Robbins et al. [45] studied the effects of pure sugar and crude sugar on the dry matter of potato tubers and found that the significant effect of crude sugar on the dry matter growth of potatoes was mainly due to the presence of VB6 in it. Liu et al. [46] studied the model plant *Arabidopsis thaliana* and found that the inhibition of ammonium oxidation stress on root growth was related to the formation of ROS in roots and that the biosynthesis of VB6 played an important role in the process of removing ROS to protect roots from stress. PLP can also balance the concentration of K+ and Na+ in the body by adjusting the activity of the ion pump and also protect plants from some environmental stress factors such as high salt and osmotic pressure [47]. In summary, combined with the results in this study that vitamin B6 metabolism is significantly up-regulated in both MDT and HDT groups, it can be concluded that this pathway plays a very positive and stable role in *L. barbarum*’s exposure to drought stress. It can be regarded as one of the pathways of *L. barbarum’s* signature rise under drought stress and its upregulated metabolites can be used as signature metabolites to reflect stress situations.

*L. barbarum* leaves also have certain medicinal properties and are rich in vitamins, linoleic acid, sulfonic acid, and amino acids [48]. The dried buds and leaves of *L. barbarum* taste mellow, fresh, and sweet, so it is also made into *L. barbarum* tea for consumption [49]. In this study, it was also found that moderate drought stress significantly upregulated taurine content in taurine and hypotaurine metabolism but, with the intensification of stress, the content decreased rapidly (Figure 9B). Taurine, known as the “ROS scavenger”, is a potent antioxidant and is also involved in the regulation of osmosis within plant cells. It is also involved in the supply and regulation of energy and helps plants resist stress [50]. This conclusion can not only designate taurine as the biomarker sensitive to drought degree in *L. barbarum* from the perspective of plant physiology but also provide a feasible reference for improving the medicinal value of *L. barbarum* leaves, that is, appropriate drought stress can promote the accumulation of its effective components. Taurine content can indicate suitability for drought stress.

In the isolated comparison between the HDT and MDT groups, the MDT showed significant upregulation of pathways, notably phenylalanine metabolism and cutin, suberine, and wax biosynthesis (Figure 7D). Phenylalanine, a precursor to a variety of secondary metabolites, including phenolic compounds, lignin, and certain plant hormones, is pivotal under drought stress [51]. Plants typically increase the synthesis of phenolic compounds to bolster their antioxidant defenses and protect against oxidative damage [52]. The enhanced synthesis of lignin may reinforce the mechanical strength of plant tissues, safeguarding against physical damage induced by drought [53]. Plant hormones such as auxins and gibberellins, synthesized via the phenylalanine metabolic pathway, regulate growth and development to adapt to arid conditions [54]. Cutin, a hydrophobic substance on the leaf surface, helps reduce water evaporation [55]. Linoleic acid, an unsaturated fatty acid involved in membrane lipid synthesis, aids in maintaining the fluidity and integrity of plant cell membranes under drought stress [56]. Increased wax biosynthesis may contribute to a more effective protective layer, minimizing non-stomatal water loss and enhancing water use efficiency [57]. In summary and in combination with the analysis results obtained from the comparison with the control, it is indicated that *L. barbarum* primarily employs metabolic pathways that enhance water use efficiency, sustain cellular osmotic balance, and eliminate ROS to counteract stress. In contrast, under severe stress, the plant appears to adopt a more enduring adaptation strategy, initiating adjustments in its energy metabolism and the redistribution of nutrients. This suggests a dynamic response to varying levels of drought stress, with the plant fine-tuning its metabolic processes to optimize survival and growth.

The metabolic mechanism of the *L. barbarum* variety “Ningqi 7” to drought stress was analyzed by metabonomics in this study, which laid a foundation for further understanding of the molecular mechanism of *L. barbarum* drought resistance. However, plant metabolism is a very complex network, which can be further studied in the future to (1) systematically elucidate the molecular response network of *L. barbarum* to drought stress through multi-omics techniques such as transcriptome and proteome; (2) allow the identification and validation of key differential metabolites and their specific functions in drought resistance to provide molecular markers for breeding; and (3) regulate the key drought resistance metabolic pathways of *L. barbarum* and improve its drought resistance performance in combination with metabolic engineering methods.

## 4. Materials and Methods

### 4.1. Source of the Tested L. barbarum Plant Specimens

The experimental material consisted of the main cultivar “Ning Qi No. 7” of *L. barbarum*, originating from Jinghe County, Xinjiang, China (82.64° E, 44.54° N). A robust *L. barbarum* plant, approximately seven years old, was selected and its semi-hardwood branches were cut into segments approximately 8 cm in length for propagation through softwood cutting. After approximately 40 days, 50 healthy, pest-free, and uniformly growing seedlings were selected and transplanted into plastic pots with dimensions of 32 cm × 28 cm, with one plant per pot. The soil used for cultivation was native sandy soil from the agricultural fields of the original habitat. Prior to the drought stress treatment, a uniform water control process was conducted to ensure adequate water supply, followed by the implementation of drought stress.

### 4.2. Drought Stress Treatment

We established three moisture treatments: the control group CK (with soil moisture content at 70% to 75% of field capacity), the moderate drought stress group MDT (40~45% FC), and the severe drought stress group HDT (10~15% FC). Each treatment had three replicates, with each replicate consisting of three pots, totaling 18 pots. The pots were weighed daily and water was supplemented as required to maintain the soil moisture content at the levels designated in the experimental design. All treatments were cultivated in a greenhouse, with a stress duration of 21 days. Following the stress period, tender stems and leaves from different plants at the same positions (the 3rd, 5th, 7th, and 9th nodes) were selected, mixed, and divided into two parts. One part was used for the determination of physiological indices in the laboratory and the other part was flash-frozen in liquid nitrogen and sent to Wekemo Tech Group Co., Ltd. (Shenzhen, China) for metabolomic analysis.

### 4.3. Determination of Physiological Parameters of L. barbarum Leaves

After harvesting the leaves, promptly measure the fresh weight of the leaves. Subsequently, immerse the leaves in distilled water to allow for complete rehydration for 48 h. Remove the leaves, pat dry the surface moisture, and measure the turgid weight. Then, dry the leaves at 65 °C to a constant weight and determine the dry weight. The relative water content (RWC) is calculated using the following formula [58]:

RWC (%) = (fresh weight − dry weight)/(turgid weight − dry weight) × 100%.

The dry matter ratio is calculated as [59]

Dry weight ratio (%) = (dry weight/fresh weight) × 100%

In order to avoid the difference in metabolite and enzyme contents in leaves under the same mass caused by the difference in water content in leaves under drought stress, the actual fresh leaf sampling amount of each treatment was corrected according to the dry weight ratio of 0.1 g.

Actual fresh leaf sampling (g) = 0.1 g/dry weight ratio

Grind the fresh leaves, which have been weight-adjusted for different treatments, in liquid nitrogen and then add 3 mL of 100 mM PBS buffer solution to create a homogenate. Uniformly use the H_2_O_2_ content, SOD (Superoxide dismutase), POD (Peroxidase), and CAT (Catalase) activity assay kits produced by Nanjing Aoqing Biotechnology Co., Ltd. (Nanjing, China) and measure the relevant parameters according to the instructions.

### 4.4. UPLC-MS Detection of L. barbarum Leaves

After slowly thawing *L. barbarum* leaf samples at 4 °C, take a fresh leaf sample, which has been weight-corrected to 0.5 g dry weight, and add it to pre-cooled methanol/acetonitrile/water solution (2:2:1, *v*/*v*). Mix by vortexing and then subject the mixture to low-temperature ultrasonication for 30 min. Allow the mixture to stand at −20 °C for 10 min, followed by centrifugation at 14,000× *g* at 4 °C for 20 min. Collect the supernatant and dry it under a vacuum. For mass spectrometry analysis, redissolve the dried sample in 100 μL of acetonitrile/water solution (acetonitrile: water = 1:1, *v*/*v*), vortex to mix, and centrifuge again at 14,000× *g* at 4 °C for 15 min. Finally, take the supernatant for injection and analysis.

The samples were chromatographically resolved using an Agilent 1290 Infinity LC (Thermo, Waltham, MA, USA) HILIC column, adhering to the protocols established by Wekemo Tech Group Co., Ltd. (Shenzhen, China). The column was maintained at a temperature of 25 °C with a flow rate set to 0.5 mL/min and a sample injection volume of 2 μL was employed. Throughout the analytical process, the samples were kept in a 4 °C automated sample injector. To mitigate the impact of instrument detection signal variability, the samples were analyzed in a randomized sequence. Quality control (QC) samples were interspersed within the sample queue to oversee and appraise the system’s stability and the dependability of the experimental data. Post-separation, via UHPLC, the samples underwent mass spectrometric analysis on a Triple TOF 6600 mass spectrometer (AB SCIEX, Framingham, MA, USA). Both the primary and secondary spectra of the samples were detected in positive and negative ion modes, respectively, utilizing electrospray ionization (ESI).

### 4.5. Metabolomics Data Analysis

The original data were converted into the mzXML format by ProteoWizard (v 3.0) and then XCMS (v1.41.0) software was used for peak alignment, retention time correction, and peak area extraction. The metabolite structure of the data extracted by XCMS was first identified and the metabolite content was then standardized for correction. In-sample correction was first performed, that is, the abundance of all features in the sample was divided by the median abundance of the sample. Then, the abundance matrix correction was carried out, that is, log conversion was performed for all the abundance values. Finally, the internal feature correction was carried out, that is, the abundance of all samples corresponding to the feature is subtracted by the mean abundance of the feature and then divided by the standard deviation of the abundance of the feature. The final metabolite content table for analysis was obtained (Table S2).

### 4.6. Data Visualization and Statistical Analysis

Differences in physiological parameters of *L. barbarum* leaves were analyzed by one-way ANOVA using GraphPad Prism (V 9.3.0) and plotted. We conducted a suite of analyses on the metabolomic data utilizing the Wekemo Bioincloud platform (https://www.bioincloud.tech/task-meta, accessed on 5 June 2024). The analyses included Principal Component Analysis (PCA) to reduce dimensionality and visualize data variability. To discern metabolic differences among groups, we employed Orthogonal Partial Least Squares Discriminant Analysis (OPLS-DA) [60]. The model’s reliability was ascertained through permutation testing, with 1000 iterations generating a random distribution of the Q2 parameter. A cutoff value of 0.5 was applied to validate the model. Variables were selected based on their contributions to the model, with a Variable Importance in Projection (VIP) score greater than one. Additionally, differential metabolites were identified through univariate statistical analysis using a t-test with a significance threshold of *p* < 0.05. Subsequently, we leveraged the MetaboAnalyst 6.0 platform (https://www.metaboanalyst.ca, accessed on 5 June 2024) to perform pathway enrichment analysis and metabolic pathway analysis on the differential metabolites [61].

## 5. Conclusions

In this study, the metabolic response mechanism of the *L. barbarum* variety “Ningqi 7” under different drought stress was revealed by using UPLC-MS analysis and antioxidant enzyme activity. Moderate drought stress (40–45% FC) induces *L. barbarum* to achieve short-term adaptation through the up-regulation of arginine, proline, phenylalanine, and wax metabolism to accumulate osmoregulatory substances, increase the antioxidant capacity, and reduce water loss. Under severe drought (10–15% FC), *L. barbarum* redistributes energy and nitrogen resources to achieve long-term adaptation by regulating purine metabolism and lysine degradation. The vitamin B6 metabolic pathway is continuously upregulated throughout the drought stress process, which may play a key role in antioxidant stabilization. These results provide a basic reference for further research on drought resistance breeding and the gene function of *L. barbarum*.

## Data Availability

The data of this study can be obtained in the paper and Supplementary Data.

## Supplementary Materials

The following supporting information can be downloaded at https://www.mdpi.com/article/10.3390/plants13141935/s1. Figure S1: The peaks obtained from all experimental samples and QC samples were analyzed by PCA; Table S1: Results of metabolic pathway enrichment analysis based on the KEGG database; Table S2: Data table: Qualitative and quantitative results table of metabolites.
