# Supplementary material for: Metabolic Response of the Lycium barbarum Variety ‘Ningqi No. 7′ to Drought Stress
Source: Plants (Basel). 2024 Jul 14;13(14):1935. doi: 10.3390/plants13141935 (PMC11280180; doi:10.3390/plants13141935)
Supplement: Supplementary file 1 [file plants-13-01935-s001.zip › plants-3080960-supplementary/SupplementaryMaterials/SupplementaryMaterials.pdf]

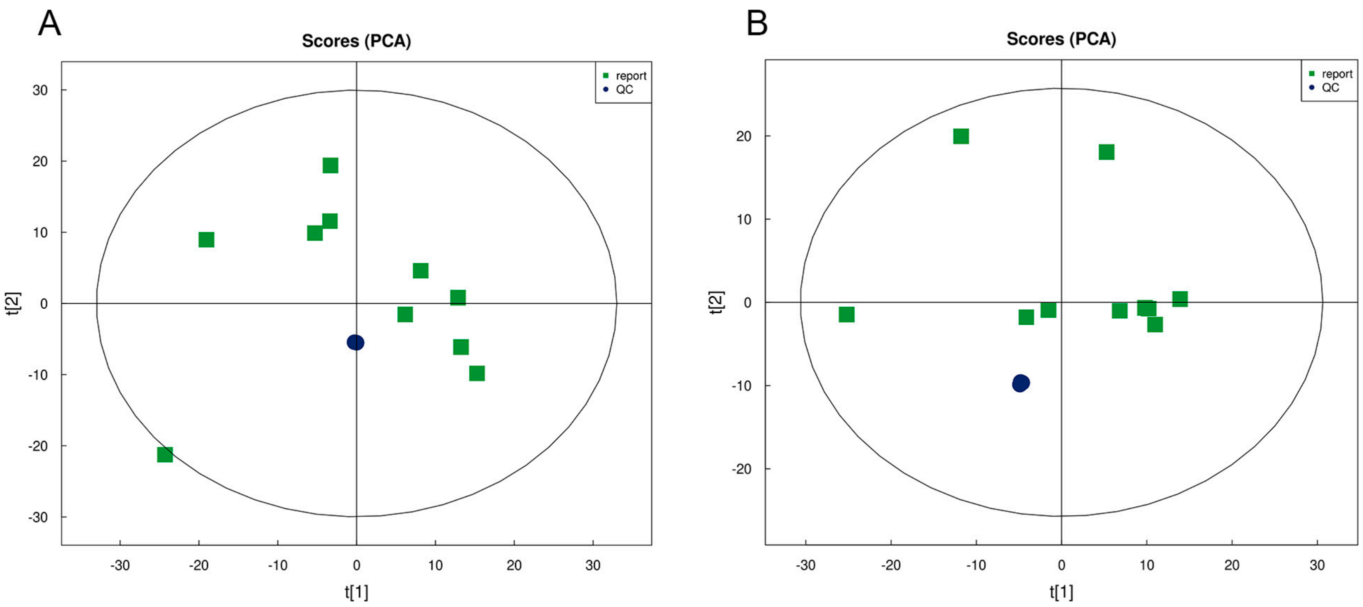

Figure S1: The peaks obtained from all experimental samples and QC samples were analyzed by PCA. Negative ion mode (A), Positive ion mode (B).

Table S1: Results of metabolic pathway enrichment analysis based on KEGG database

Comparison Group	Pathway	Total	Expected	Hits	Raw p	Impact
MDT&HDT.VS.CK UP	Arginine and proline metabolism	32	0.49067	4	0.001138	0.16965
	Vitamin B6 metabolism	12	0.184	2	0.013525	0.18095
	Phenylalanine metabolism	12	0.184	2	0.013525	0.76923
	Arginine biosynthesis	18	0.276	2	0.029652	0.13592
	Phenylalanine, tyrosine and tryptophan biosynthesis	22	0.33733	2	0.043138	0.01001
	Alanine, aspartate and glutamate metabolism	22	0.33733	2	0.043138	0.19424
	alpha-Linolenic acid metabolism	26	0.39867	2	0.05849	0.125
	Tryptophan metabolism	29	0.44467	2	0.071074	0.25191
	Taurine and hypotaurine metabolism	5	0.076667	1	0.074448	0.625
	Glycerophospholipid metabolism	38	0.58267	2	0.11332	0.12248
	Pyrimidine metabolism	41	0.62867	2	0.12861	0.02926
	beta-Alanine metabolism	18	0.276	1	0.24402	0.07143
	Purine metabolism	73	1.1193	2	0.30928	0.01268
	Pantothenate and CoA biosynthesis	25	0.38333	1	0.32257	0.02796
	Biosynthesis of various plant secondary metabolites	29	0.44467	1	0.36389	0.24
	Steroid biosynthesis	44	0.67467	1	0.49838	0.00744
MDT&HDT.VS.CK Down	Glyoxylate and dicarboxylate metabolism	29	0.13533	2	0.00714	0.06012
	Vitamin B6 metabolism	12	0.056	1	0.054781	0.06667
	Arginine biosynthesis	18	0.084	1	0.081192	0.08544
	Citrate cycle (TCA cycle)	20	0.093333	1	0.089855	0.11571
	Starch and sucrose metabolism	22	0.10267	1	0.098447	0.0174
	Alanine, aspartate and glutamate metabolism	22	0.10267	1	0.098447	0.32374
	Glutathione metabolism	26	0.12133	1	0.11542	0.05046
HDT.VS.MDT UP	Purine metabolism	73	0.38933	2	0.054079	0.05835
	Lysine degradation	20	0.10667	1	0.10205	0.16667
	Valine, leucine and isoleucine degradation	37	0.19733	1	0.1815	0.00991

	Fatty acid degradation	37	0.19733	1	0.1815	0.02723
	Pyrimidine metabolism	41	0.21867	1	0.19927	0.13906
HDT.VS.MDT Down	Phenylalanine metabolism	12	0.048	1	0.047127	0.34615
	Cutin, suberine and wax biosynthesis	18	0.072	1	0.069987	0.125
	Thiamine metabolism	22	0.088	1	0.084972	0.00578
	Phenylalanine, tyrosine and tryptophan biosynthesis	22	0.088	1	0.084972	0.00851
	Terpenoid backbone biosynthesis	30	0.12	1	0.11434	0.03751
	Flavonoid biosynthesis	47	0.188	1	0.17414	0.10333
